# Improvement of Planning Skills in Children With Autism Spectrum Disorder After an Educational Intervention: A Study From a Mixed Methods Approach

**DOI:** 10.3389/fpsyg.2019.02824

**Published:** 2019-12-17

**Authors:** Elena Escolano-Pérez, Marian Acero-Ferrero, Mª Luisa Herrero-Nivela

**Affiliations:** Department of Psychology and Sociology, University of Zaragoza, Zaragoza, Spain

**Keywords:** mixed methods, systematic observation, autism spectrum disorder, children, executive functions, planning, lag sequential analysis, educational practice

## Abstract

The literature confirms that individuals with autism spectrum disorder (ASD) have planning deficits. However, few interventions have targeted these deficits. The aims of this study were to: (1) show that the mixed methods approach can be useful in studying planning skills of children with ASD during and after an educational intervention; (2) assess whether the planning skills of two groups of children with ASD improved during the intervention and if this progress was maintained 1 month after completing the intervention. The groups were formed depending on each child’s severity level (SL) of ASD according to DSM-5: SL1 (requiring support) and SL2 (requiring substantial support). Each group was composed of four children. In the framework of mixed methods, we used observational methodology, which is considered as mixed methods in itself because it integrates qualitative and quantitative elements. A nomothetic/follow-up/multidimensional observational design was used. Planning skills manifested by children during the intervention were codified, as well as the scaffolding behaviors provided by the educational specialist. These skills and behaviors were also coded in one session, which took place 1 month after the intervention. Coded data of each group were submitted to prospective and retrospective lag sequential analysis. This informed of the sequential structure of planning skills performed by children in interaction with the educational specialist at the beginning and at the end of the intervention, as well as 1 month later after the intervention. The comparison of the patterns obtained in these three temporal moments allowed us to know the improvement of the two groups in the use of planning skills. Results showed that both groups improved their autonomous use of planning skills. However, SL1 group used successfully and autonomously complex planning skills, while SL2 group were unable to achieve this gain. SL2 group progressed in autonomy, but only using basic planning skills. Both groups can further improve their use of planning skills; therefore, the intervention should be adjusted to their characteristics and temporarily extended. These findings contribute to the, as yet, little studied field of intervention and assessment of planning skills in children with ASD using a mixed methods approach.

## Introduction

Autism spectrum disorder (ASD) is a neurodevelopmental disorder characterized by: (1) persistent deficits in communication and social reciprocity and (2) patterns of restricted and repetitive behavior, interests, or activities. The consideration of the disorder within a continuity of severity and involvement in each of the two domains facilitates the identification of the great symptomatic heterogeneity within the spectrum. Thus, depending on the severity of the symptoms (and therefore, the support that individuals with ASD require), Diagnostic and Statistical Manual of Mental Disorders (DSM-5; American Psychiatric Association, 2013) establishes three severity levels (SL): SL1 = “requiring support”; SL2= “requiring substantial support”; SL3= “requiring very substantial support.”

Under these diagnostic criteria, certain cognitive styles characterize a peculiar learning and a specific daily functioning in people with ASD, which could be explained by the deficit in executive functions (Ozonoff, 1995; Poljac and Bekkering, 2012). Executive functions are high-level cognitive and affective processes that direct thoughts, emotions, and behaviors during an active problem solving, especially those that require a novel approach (Carlson et al., 2013; Diamond, 2013). These set of differentiate skills are both interactive (Ozonoff, 1995) and integrate a wide range of cognitive functions such as planning, monitoring, working memory, flexibility, inhibition, and generativity (Hill, 2004b). An executive dysfunction in ASD is evidenced by numerous empirical studies. This dysfunction includes difficulties in different executive domains that extend to all areas of life and are present in all ages and SL, generating in the person a serious adaptive deterioration that affects their daily functioning (Hill, 2004a; Smithson et al., 2013; Geurts et al., 2014; Panerai et al., 2014; Demetriou et al., 2018; Vogan et al., 2018; Valeri et al., 2019).

Among all the executive deficits present in people with ASD, the one referred to planning is one of those with the highest prevalence and which generates the most difficulties in the individual’s daily life. Planning deficit implies problems to choose and implement a sequence of actions to achieve a pre-specified goal (Lezak, 2012). For such planning to be effective, this sequence of actions must be checked, evaluated, and constantly updated. However, people with ASD manifest difficulties in these processes (Hill, 2004a; Olde Dubbelink and Geurts, 2017).

The abundant literature confirming these executive deficits in ASD contrasts with the shortage of cognitive intervention programs aimed at their improvement (Russell, 1997; Ozonoff et al., 2005; Kenworthy et al., 2014; Vogan et al., 2018). Therefore, much more work is needed in order to design effective early interventions. In this regard, it has pointed out the usefulness of ecological environments in which children with ASD participate in game-based activities (Traverso et al., 2015; Rice, 2016; Hillman, 2018; Vogan et al., 2018). Playing is a child’s natural way of expression and provides opportunities for his/her development and learning. This makes games an essential tool for children’s teaching-learning process and for the systematic observation and analysis of their progress (Fasulo et al., 2017; Otsuka and Jay, 2017; Zosh et al., 2018). However, despite its importance, there are few works that carry out a valid and reliable child intervention and evaluation based on games (Salcuni et al., 2017; Hillman, 2018).

Another aspect that has also shown influence on the development of executive functions in children is social interaction (Carlson, 2009; Lewis and Carpendale, 2009; Amadó et al., 2016; Sosic-Vasic et al., 2017). Modeling, anticipation, and scaffolding are the most common social interaction strategies (Bibok et al., 2009; Hughes and Ensor, 2009; Devine et al., 2016). In this study, we focus on scaffolding. It refers to the process by which adults help, plan, and organize the activity of children so that they can carry out a task that goes beyond their skill level (Wood et al., 1976; Vygotsky, 1978). In this regard, the importance of reinforcing messages providing emotional support in favoring the performance of certain executive capacities has also been evidenced (Vandenbrouck et al., 2017). In short, the literature indicates that fundamental aspects for an effective intervention of executive functions in children with ASD are: (a) that it takes place in an ecological context, (b) where the child develops playful activities, and (c) in company of an adult who offers an adequate scaffolding.

Regarding the methodological issues for the evaluation of executive functions in children with ASD, the mixed methods approach is the most appropriate strategy as it integrates qualitative and quantitative elements. Within this perspective, the idoneous option is systematic observation since it allows to capture spontaneous behaviors as they occur in a natural context (Portell et al., 2015a,b), and therefore, is the most appropriate methodology for assessing interventions in young children in ecological environments (Anguera, 2001; Whitebread and Pino-Pasternak, 2013). Taking into account the aforementioned benefits of the children’s games, and given that playing is a ubiquitous and universal aspect of childhood, the systematic observation of a child’s behavior in a playful context offers a wealth of information and a variety of nuances that allow describing, explaining, and understanding fundamental aspects of child development and learning imperceptible with other methodologies (Escolano-Pérez et al., 2017).

Observational methodology nowadays is considered in itself as mixed methods because it integrates qualitative and quantitative elements in QUAL-QUAN-QUAL phases (Anguera et al., 2017, 2018a,b; Arias-Pujol and Anguera, 2017; Del Giacco et al., 2019; Portell et al., 2019). In a first QUAL phase an *ad hoc* observation instrument must be elaborated and data must be codified based on an order or sequence criterion, thus making it possible to capture in a natural context the spontaneous behaviors that indicate the competences under study. After, a QUAN phase follows in which the quality of the coded observational data is tested (intra-observer and/or inter-observer agreement analysis) and its analysis is carried out. Precisely because the initial dataset, which is derived from an extremely rich qualitative component, can be analyzed using different quantitative techniques suitable for qualitative data (as lag sequential analysis) a set of quantitative results (as patterns of behavior) are obtained. Finally, the interpretation of the results (considering the objective of the study and prior researches) returns the process to the QUAL phase.

Taking into account: (a) all aspects mentioned above; (b) the minimum quality criteria and fundamental indicators that should guide the intervention targeted at people with ASD (Kasari and Smith, 2013) and specifically the educational interventions for children with ASD (Bond et al., 2016; Otero et al., 2017); and (c) the general principles of intervention in executive functions (Diamond and Lee, 2011; Diamond, 2014), an educational intervention aimed at improving planning skills in children with ASD was developed. (It is described in Procedure section).

There were two objectives of this study, one methodological and one educational: (1) show that the mixed methods approach can be useful in studying planning skills of children with ASD during and after an educational intervention; (2) assess whether the planning skills of two groups of children with ASD (grouped according to their SL) improved during the intervention and if this progress was maintained 1 month after the end of the intervention.

## Methods

### Design

We applied a mixed methods approach as an ongoing method of assessment that is characterized by the integration of qualitative and quantitative elements. In this study, the integration was carried out from the “connect” option (Creswell and Plano Clark, 2011).

The observational design employed, according to the observational designs described by Anguera et al. (2001) and Anguera et al. (2018a), was Nomothetic/Follow-up/Multidimensional. It was: “nomothetic” because eight children with ASD were observed individually and their observational data were later treated jointly in two units of analysis (depending on each child’s SL of ASD according to DSM-5); “follow-up” at both the inter-sessional and intra-sessional levels because 25 sessions were observed for each child and also recorded from beginning to end of each session; and “multidimensional” because different levels of response were observed referring to the children’s planning skills and adult scaffolding behaviors that supported the observation instrument.

The systematic observation carried out was non-participative and active and behaviors observed were fully perceivable (Anguera, 2001; Bakeman and Quera, 2011).

### Participants

The final sample included eight Spanish children with ASD, male gender between 5 years 6 months and 12 years (*M* = 92.37 months; SD = 23.24). Four participants presented SL1 (requiring support) according to the DSM-5 criteria and four participants SL2 (requiring substantial support). Table 1 shows the characteristics of the participants of each group. They all received a formal diagnosis of ASD made by a multidisciplinary team according to the DSM-5 criteria for ASD. According to the current regulations in Spain, their diagnosis was additionally confirmed by at least one child psychiatrist with expertise and considerable experience in autism not associated with the current study through the ADOS-2 (Lord et al., 2000).

**Table 1:** Characteristics of the participants of each group.

Group	Participants	Age (months)	IQ	Mean Age (months)	SD Age (months)	Mean IQ	SD IQ
SL1	1	82	105	83.5	12.79	101.25	12.34
	2	94	107				
	3	92	83				
	4	66	110				
SL2	5	144	54	101.25	29.77	62	10.46
	6	76	72				
	7	97	52				
	8	88	70				

The inclusion criteria for participation in the study were: (1) confirmed diagnosis of ASD (DSM-5); (2) age 5–12; (3) sufficient verbal skills: score less than 5 in each of the three dimensions of the Autism Spectrum Inventory communication and language scale (Autism Spectrum Inventory, IDEA) (Rivière, 2002); (4) informed consent of the students’ parents authorizing their participation in the study. Moreover, exclusion criteria were: (1) IQ ≤ 49; (2) diagnosis of physical disability; (3) co-morbidity with a psychiatric disorder.

The information referring to the inclusion criteria (1), (2), and (3) and the exclusion criteria (2) and (3) were provided by the educational guidance team of the school to which each participant attended. For the information referring to the exclusion criterion (1), the Wechsler Intelligence Scale for Children-Fourth Edition (WISC-IV) was administered in the Spanish version (Corral et al., 2005).

Participants were treated according to the international ethical standards and Spanish Organic Law 15/1999 of December of Protection of Personal Data (Spanish Government, 1999). Research was evaluated and approved by the Education Doctoral Program Academic Commission of Zaragoza University and by the management teams of the schools attended by the participants. No ethics special approval was required for this research since the Spanish public education system and national regulations require no such approval.

### Instruments

#### Observation Instrument

**Figure 1:**
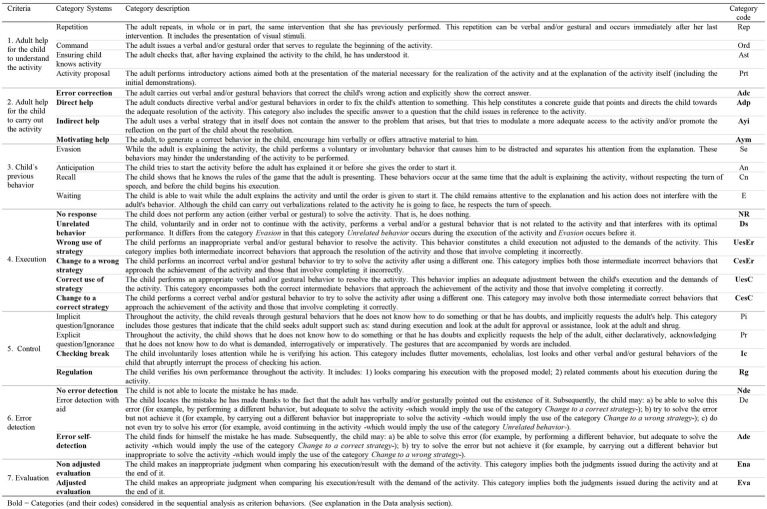
Observation instrument.

An observation instrument was built *ad hoc* to allow us to observe the children’s actions indicating planning skills, in addition to adult scaffolding behaviors. Since our study was multidimensional, the observation instrument combined a field format with category systems. It contained seven criteria and each criterion was broken down into an exhaustive and mutually exclusive category system. The instrument was developed according to: (a) previous recordings of children of similar characteristics to those of the participants. In these recordings, children were solved habitual tasks of their daily life and whose resolution required the use of planning skills; (b) the theoretical framework related to the executive function of planning, especially in ASD (Zelazo et al., 1997; Hill, 2004a; Ward and Morris, 2005; Olde Dubbelink and Geurts, 2017) and scaffolding (Wood et al., 1976; Vygotsky, 1978; Bibok et al., 2009; Bernier et al., 2010); (c) observation instruments used by other researchers to capture, among other issues, planning skills in children (Whitebread et al., 2009; Escolano-Pérez and Sastre-Riba, 2010). The constructed observation instrument is presented in Figure 1.

Criteria 1 and 2 were related to the adult behaviors and the remaining provided information about child behaviors. Each criterion was broken down into categories that implied directly observed behaviors during the intervention sessions. Criterion 1 (*Adult help for the children to understand the activity*) was broken down into four categories. In category *Repetition,* the adult updated the information to the child if she detected working memory fails. *Command* indicated the help offered by the adult when child showed inhibitory difficulty to resist his impulse to start the task before the adult ordered it. Due to comprehension problems in ASD, the category *Ensuring child knows the activity* revealed behavior executed by adult to check if the child had understood the activity. *Activity proposal* meant that the adult was explaining the activity to the child. In this category, the adult helped the child to focus attention to the explanation of the activity. Criterion 2 (*Adult help for the child to carry out the activity*) was broken into four categories. This criterion was referred to different scaffolding behaviors that adult provided to the child to help him during the execution of the activity. The categories of this criterion was graduated according to levels of support, from those that involved more help to the child (*Error correction*) to those that implied less support to him (*Motivating help*). Criterion 3 (*Child’s previous behavior*) was broken down in four categories. *Evasion* showed the child’s inability to maintain his attention while the adult explained the activity. *Anticipation* referred to the child’s difficulty in inhibiting their impulses. *Recall* was directly related to the use of working memory. *Waiting* showed an adequate inhibitory process. *Anticipation* indicated an inadequate inhibitory process. Criterion 4 (*Execution*) showed the child’s behaviors during the resolution of planning activities. It was broken down in six categories. *No response* denoted the child inability to generate strategies to resolve the activity. *Unrelated behavior* involved child failures in attention. *Wrong use of strategy* indicated child used incorrectly his executive process and committed a mistake. *Change to a wrong strategy* was linked to an inadequate cognitive flexibility. Although the information was updated, it was not been done properly. *Correct use of strategy* entailed the child used correctly his executive process during the resolution of the activity. *Change to a correct strategy* was related to an adequate cognitive flexibility: the child was able to update, to check and to make his actions more adaptable to achieve a specific goal. Criterion 5 (*Control*) was broken down in four categories. *Implicit question/Ignorance* indicated a break in the sequence of actions. In this category, child realized he was stuck and he indirectly asked for help. *Explicit question/Ignorance* also indicated a break in the sequence of actions. Child also realized he was stuck but in this case, he verbally asked for help. *Checking break* implied a disruption during the child verification process. The child was unable to hold his attention until the end of this process. *Regulation* showed that the child verified his actions to solve adequately the activity. Criterion 6 (*Error detection*) was broken down in three categories. *No error detection* meant failures in planning (particularly as regards updating process). *Error detection with aid* implied that these failures persisted but the child could correct it with adult help. *Error self-detection* showed a greater functioning in updating process due the child was able to realize his mistake. Criterion 7 (*Evaluation*) was broken down in two categories. *Non adjusted evaluation* involved deficits in planning. The child was unable to evaluate properly the result of his actions. *Adjusted evaluation* involved the child was able to evaluate adequately the result of his actions.

#### Recording Instruments

A digital video camera was used to record the infant and adult action in each session of intervention.

For the coding process we used LINCE (v.1.2.1) (Gabín et al., 2012). This software can be downloaded free from http://lom.observesport.com/.

#### Data Analysis Instrument

The software GSEQ5, v.5.1 (Bakeman and Quera, 2011) was used for the data quality check (intra- and inter-observer reliability) and the lag sequential analysis. It can be downloaded for free^1^.

### Procedure

The management team of 10 schools from a northeastern city of Spain where students with ASD can attend were informed about the research. Six schools were interested in participating in the research. After informing the parents, collecting their informed consent and applying the inclusion and exclusion criteria in the sample, nine potential participants were obtained: five presented SL1 and four presented SL2. Since, one participant did not attend all intervention sessions because of illness, the final sample was configured by eight participants (4 of each SL).

**Figure 2:**
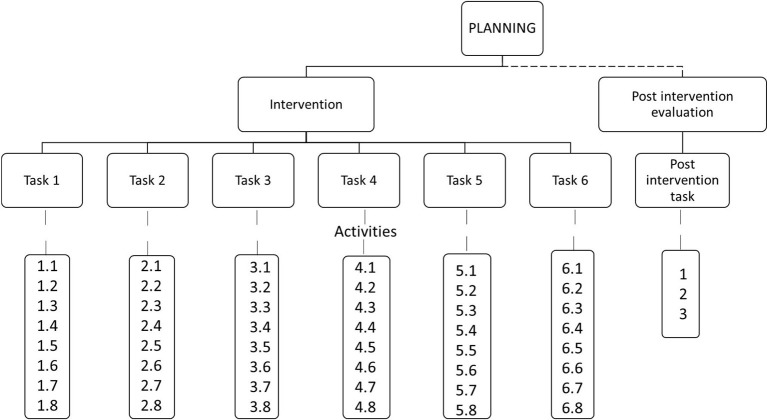
Structure of intervention and post intervention evaluation in planning skills.

Each of the participants individually received the intervention designed to improve planning skills. This intervention (Figure 2) consisted of six tasks whose resolution required the implementation of planning skills. All tasks were oriented to real life and had a functional nature. In task 1, children were asked to order a sequence of facts showed out of order in different pictures. Task 2 consisted that children had to outline an action plan in order to find the maze’s right exit avoiding the obstacles along the way. In task 3, children had to select the necessary ingredients to elaborate a specific sandwich. Task 4 required children to choose among several dishes alternatives, the food they needed to prepare a whole menu. In task 5, children had to pack up in a suitcase with forethought all the clothes they will need to go to the beach on summer. In task 6, children were asked to program in a map their visit route to a village while visiting several places (school, library, fire station…). Children were instructed to follow some rules (i.e., do not cross the walls, walk only on the sidewalk…) and to avoid using the same route twice. Each task consisted of eight playful activities of increasing difficulty. Thus, the whole intervention was composed of 48 activities.

The intervention for each participant took place over 24 sessions of half an hour each. In each session, two activities were carried out. The intervention lasted 3 months, with 2 sessions per week on non-consecutive days.

One month after the end of the intervention, a session was held in which each participant was given one post intervention evaluation task similar to those of the intervention. More exactly, in this task, children are asked to organize the correct order of different clothes to dress up a puppet step by step. This task consisted of three activities of increasing difficulty. The purpose of this post intervention evaluation task was to check if the level of planning skills of the participants was maintained over time.

All sessions were carried out at the children’s schools by the same educational specialist and were recorded for later viewing and analysis.

The video recordings were imported into the LINCE software and coded by an expert observer in executive functions and ASD using the *ad hoc* observation instrument.

#### Control of the Quality of the Data

The quality of the observational data was controlled using two guidelines (Anguera, 2003): (a) Qualitative: consensual agreement was used in the first session to be coded for each participant (therefore, a total of eight sessions) by three expert observers in observational methodology, ASD and executive functions; (b) Quantitative: (b1) intra-observer reliability was calculated in three sessions for each child: one belonging to task 1, another to task 6 (both sessions randomly chosen) and the post intervention task session. We selected sessions of these three tasks because these tasks were the ones analyzed in this study, as will be explained later; (b2) inter-observer reliability was also calculated in three sessions for each child: one of task 1 and one of task 6 (randomly selected sessions but taking care that they were different from those used for the calculation of intra-observer reliability) and the post intervention task session. Cohen’s kappa coefficient (Cohen, 1960) was calculated using software program GSEQ5, v.5.1. All results obtained are between 0.84 and 1.00, which according to the scale suggested by Landis and Koch (1977) corresponds to a very good agreement. Therefore, the quality of the observational data obtained was excellent.

### Data Analysis

#### Lag Sequential Analysis

Lag sequential analysis was proposed by Bakeman (1978), and subsequently extended by Bakeman and Gottman (1986), Bakeman and Quera (2011), and Quera (2018). It is a highly effective data analysis technique for analyzing datasets compiled from observation that contain sequences of behaviors that are coded using an *ad hoc* observation instrument. It allows to detect those patterns of behavior, which occur with greater probability than would be predicted by chance. That is, starting with a behavior considered as a pattern generator (criterion or given behavior), and that tends to be chosen according to the objectives of each study, it looks for which other behaviors appear associated with the same with a probability greater than mere chance (conditional behaviors), both later (positive lag: +1, +2, etc.), and earlier (negative lag: −1, −2, etc.). In this way, prospective and retrospective patterns are obtained. In our investigation, these patterns allowed us to identify the sequential structure of the behaviors that the participants performed in interaction with the adult at the beginning and end of the intervention, as well as 1 month after the end of the intervention. The comparison of the patterns obtained in these three temporary moments allowed us to know their progress in the use of planning skills.

This technique has been widely used in studies that analyze sports behavior, but its use in studies in the educational field is somewhat sparse (Herrero-Nivela and Pleguezuelos, 2008; Santoyo et al., 2017; García-Fariña et al., 2018; Escolano-Pérez et al., 2019). Especially, scarce are the studies with ASD students in which this data analysis technique has been used (Canal and Rivière, 1993; Rodríguez-Medina et al., 2018). We are unaware of works in which this data analysis technique has been used to evaluate planning skills in ASD children, a novel issue that is addressed in this work.

In our study, the records of the participants were joined according to their SL (SL1 or SL2) and the task to which they corresponded.

In each group of participants (SL1 and SL2), three tasks were analyzed: task 1 and task 6 (as they were the tasks that best allowed us to know the improvement of each group during the intervention when comparing the planning skills used at the beginning and at the end of the intervention) in addition to the post intervention task (this reflected the possible maintenance of the improvement after 1 month without intervention). To perform lag sequential analysis for each group and task: (1) the same criteria behaviors were chosen: those categories considered most relevant depending on the objective of the study given that they play a central role in planning skills and the resolution of daily activities. Specifically, the following categories—appearing alone or co-occurring with others—were chosen (categories indicated in bold in Figure 1): (1a) of those referring to participants: (i) all of criterion 4 *Execution*: *No response* (*NR*), *Unrelated behavior* (*Ds*), *Wrong use of strategy* (*UesEr*), *Change to a wrong strategy* (*CesEr*), *Correct use of strategy* (*UesC*) and *Change to a correct strategy* (*CesC*), as they indicated the accuracy of the response/answer that the participants issued during the execution of the activity as well as their flexibility to change the strategy; (ii) of criterion 5 *Control*, categories *Checking break* (*Ic*) and *Regulation* (*Rg*) given that they showed the existence of check behaviors of the participants on their own action and its quality; (iii) of criterion 6 *Error detection*, categories *No error detection* (*Nde*) and *Error self-detection* (*Ade*) as they showed the ability of the children to update their own activity during the performance of the task and therefore to detect or not the errors produced; (iv) all categories of criterion 7 *Evaluation*: *Non adjusted evaluation* (*Ena*) and *Adjusted evaluation* (*Eva*) because the evaluation informs about the children’s ability to distinguish whether an objective has been achieved or not, and examine their execution/result comparatively with the demands of the task; (1b) of those categories referring to the adult, all ones of the observation instrument belonging to criterion 2 *Adult help for the child to carry out the activity: Error correction* (*Adc*), *Direct help* (*Adp*), *Indirect help* (*Ayi*), and *Motivating help* (*Aym*). They were chosen because they involved scaffolding strategies that provided support for the children in carrying out the tasks; (2) as given behaviors, all the categories that make up the observation instrument (Figure 1) were considered; (3) lag sequential analysis were performed prospectively (from +1 to +5 lags) and retrospectively (from −5 to −1 lags); (4) the level significance was set at *p* < 0.05.

## Results

Of all the patterns obtained (61 retrospective and 45 prospective patterns in SL1 group, 34 retrospective and 35 prospective patterns in SL2 group), the most relevant for this study are described below. They are the most relevant because: (a) are generated by the children or (b) they are generated by the adult but contain infant’s and adult’s behaviors. Therefore, these patterns are those allow us to know the use of infant’s planning skills. Consequently, the patterns generated by the adult that only contain adult’s behaviors are not relevant and they are not described.

**Figure 3:**
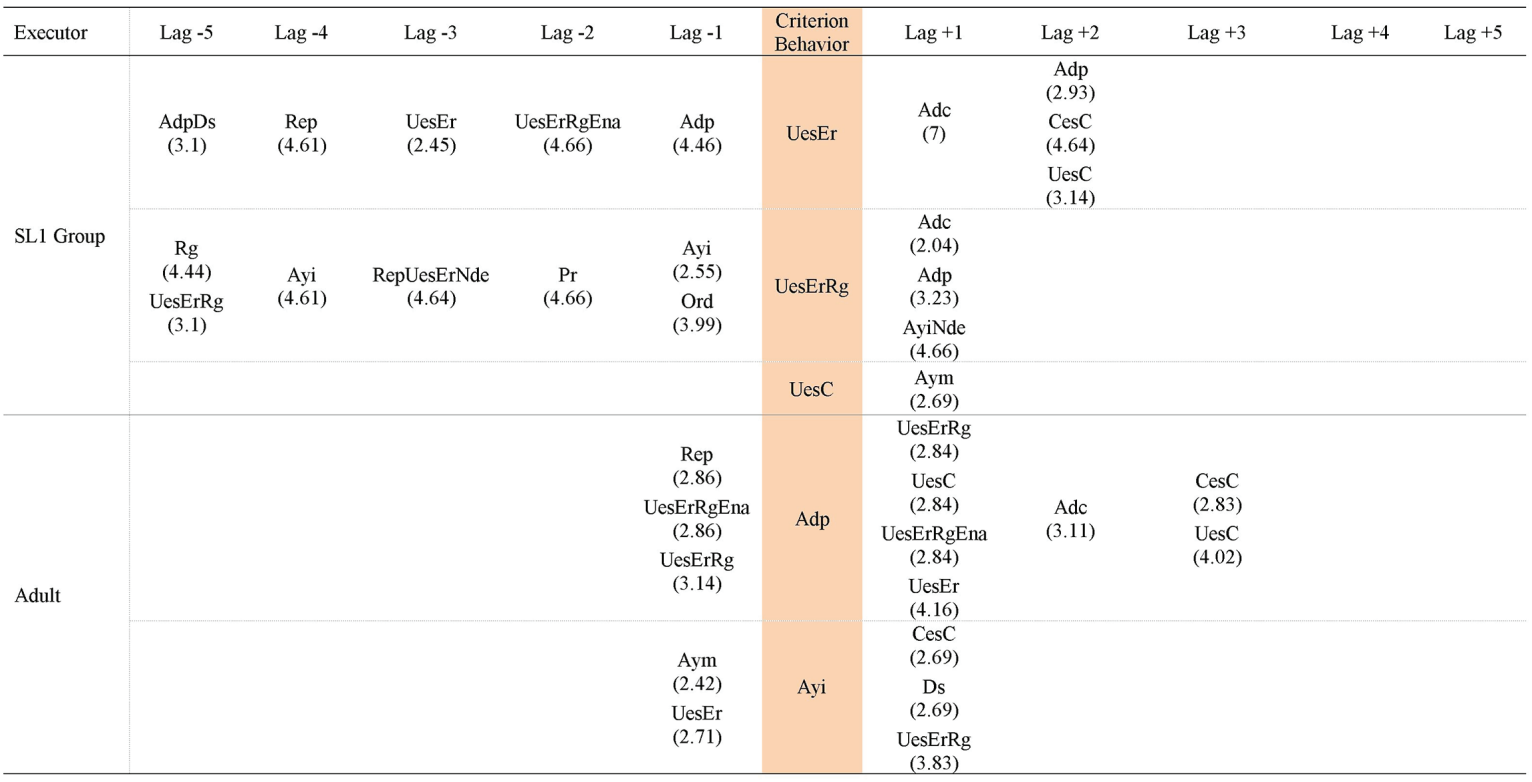
Prospective and retrospective patterns obtained in SL1 group. Task 1.

**Figure 4:**
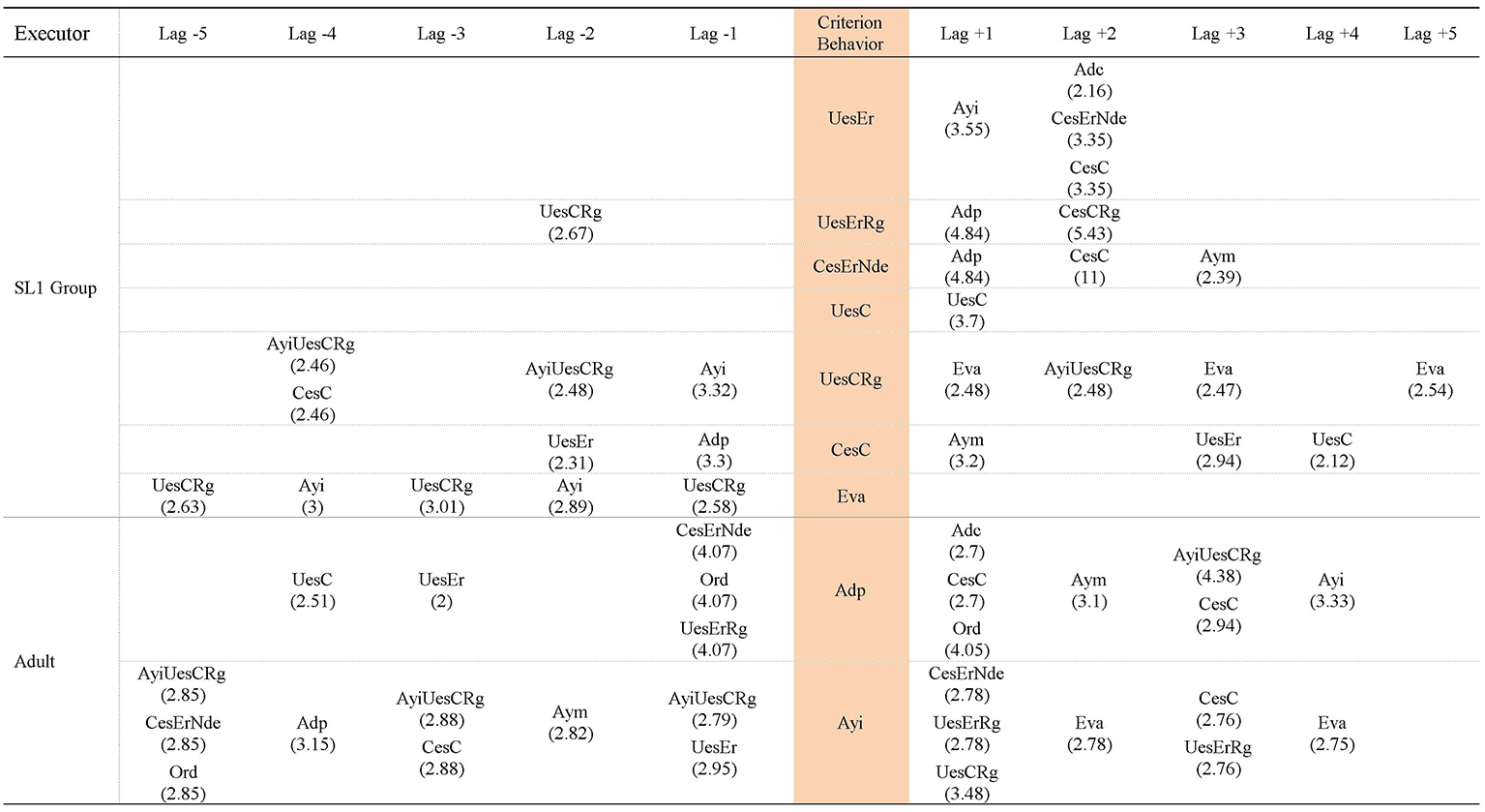
Prospective and retrospective patterns obtained in SL 1 group. Task 6.

**Figure 5:**
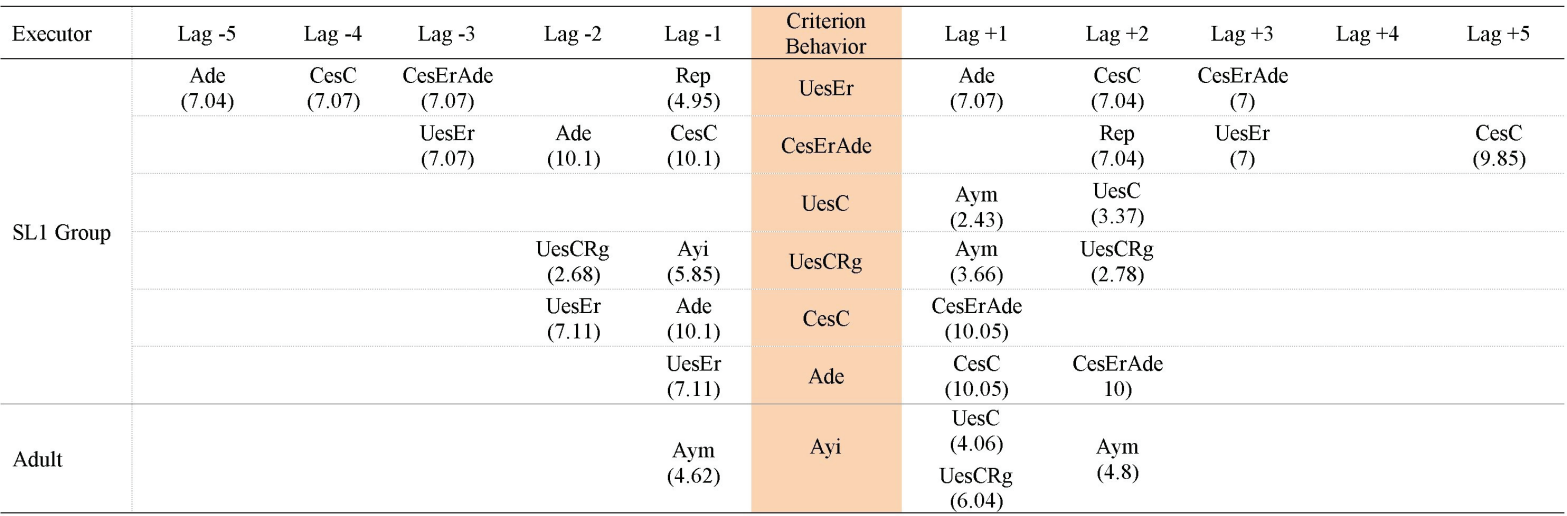
Prospective and retrospective patterns obtained in SL 1 group. Post intervention task.

**Figure 6:**
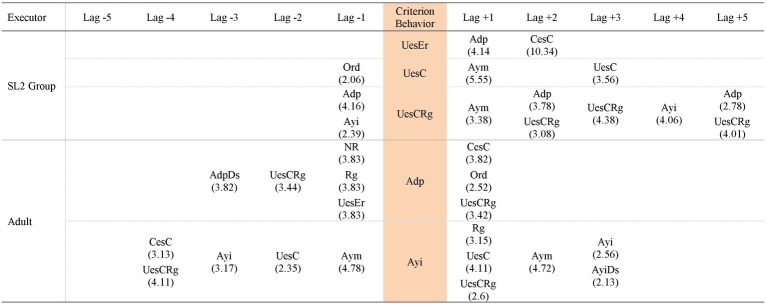
Prospective and retrospective patterns obtained in SL2 group. Task 1.

**Figure 7:**
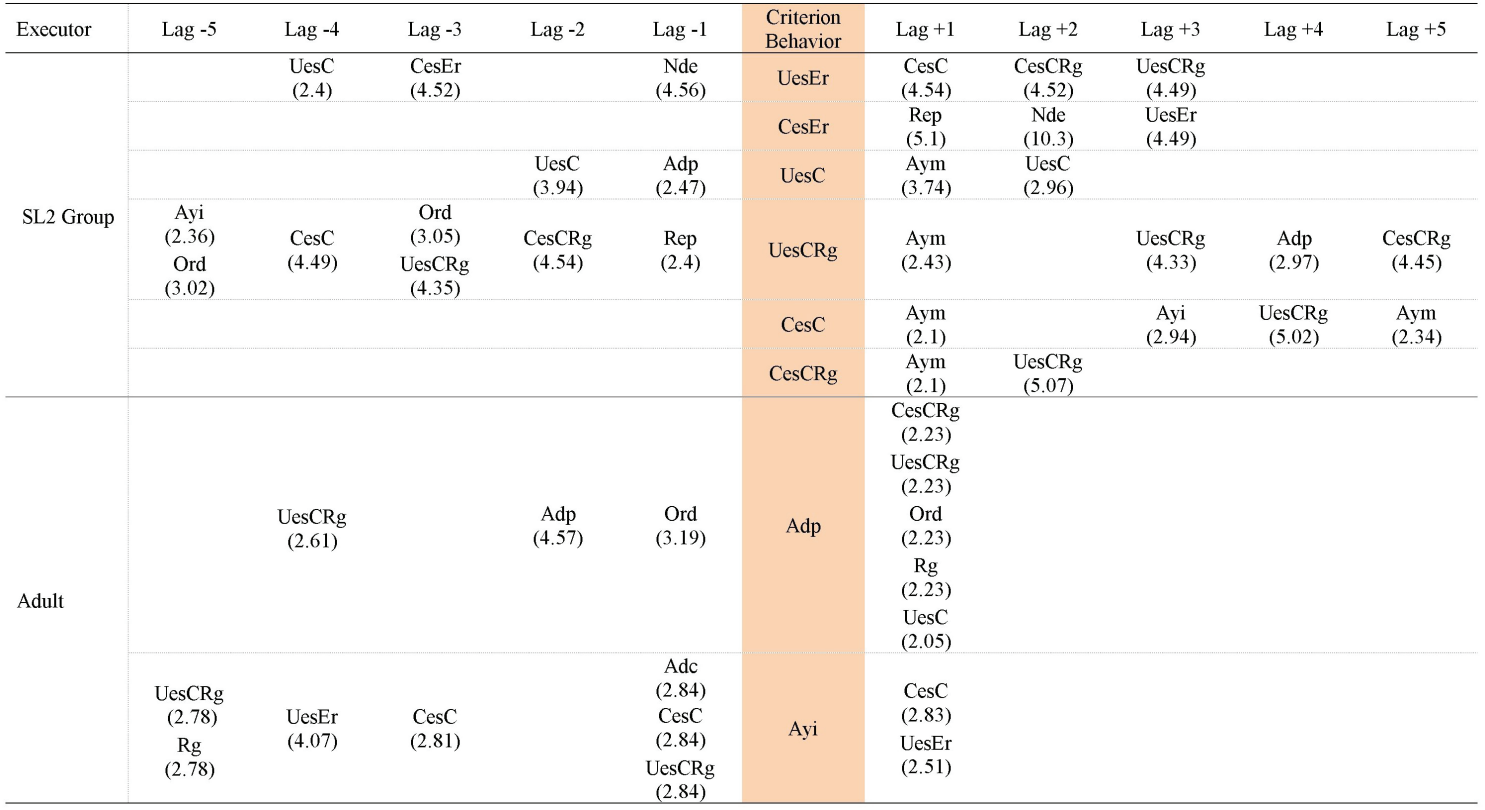
Prospective and retrospective patterns obtained in SL2 group. Task 6.

**Figure 8:**
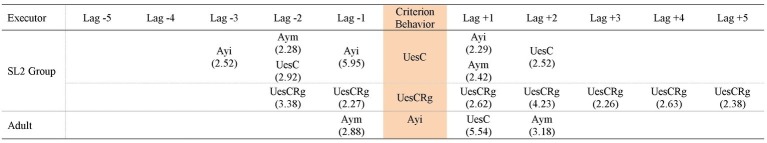
Prospective and retrospective patterns obtained in SL2 group. Post intervention task.

**Figure 9:**
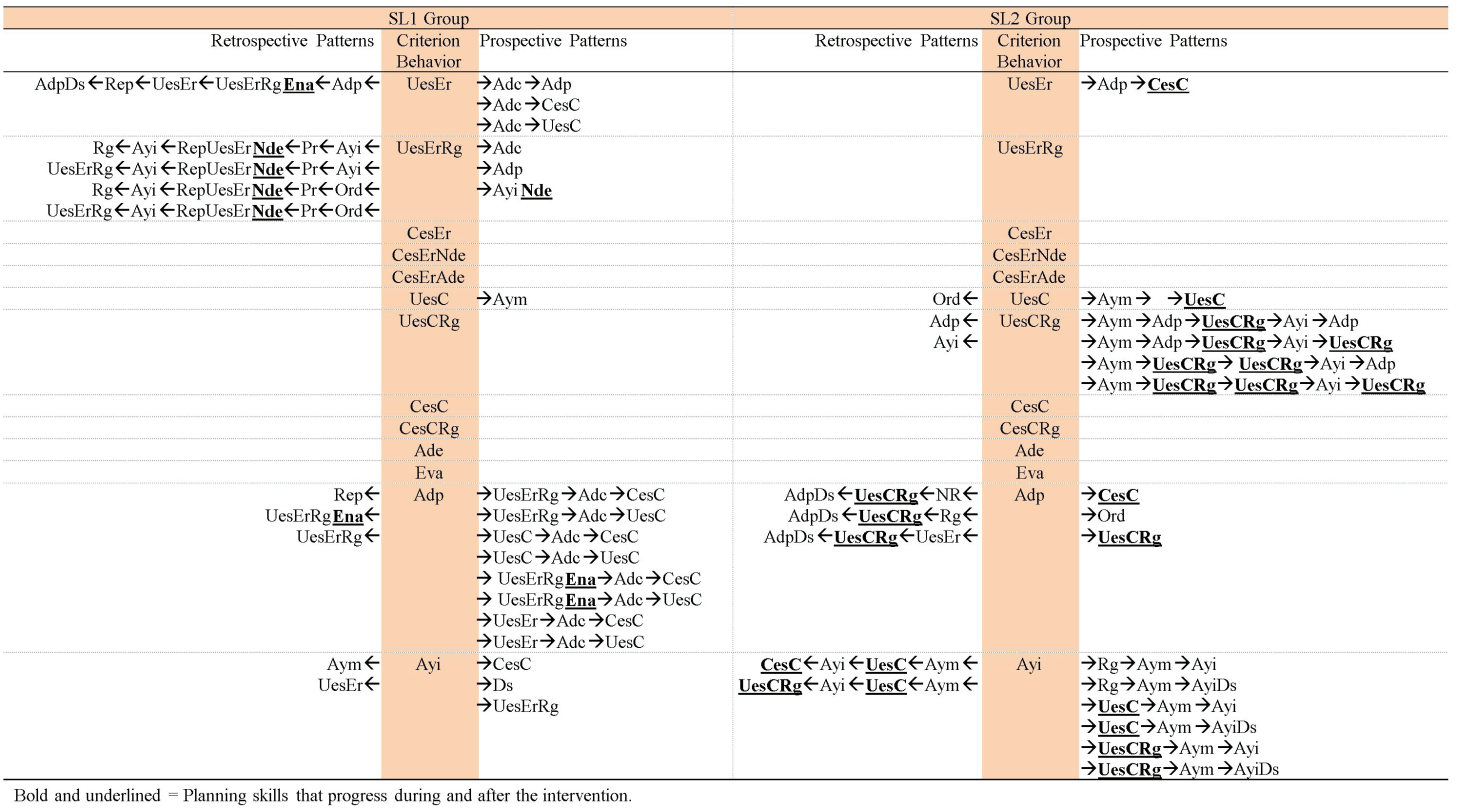
Comparison of the patterns obtained in each group in task 1.

**Figure 10:**
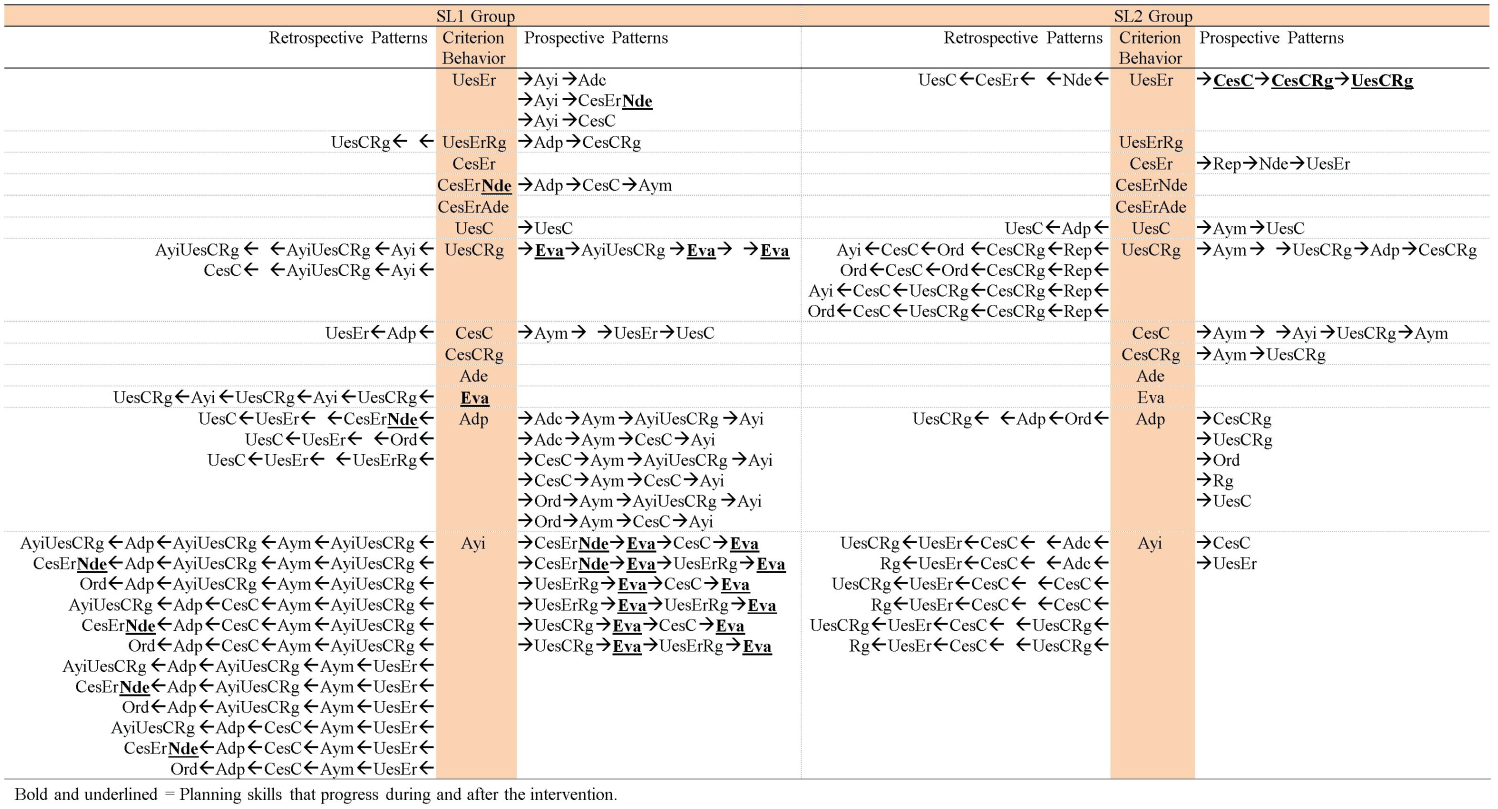
Comparison of the patterns obtained in each group in task 6.

**Figure 11:**
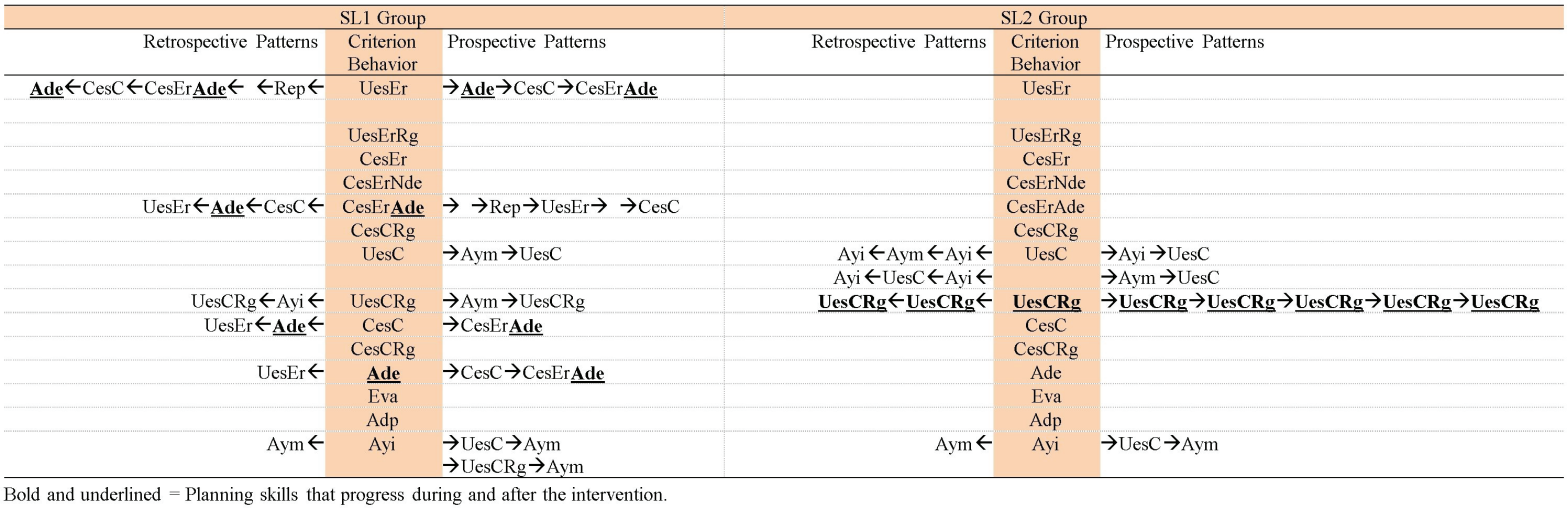
Comparison of the patterns obtained in each group in the post intervention task.

Hence, the patterns generated by each group of participants in each task analyzed are presented, in addition to the patterns generated by the adult that included behaviors of the participants of each group in each task. Thus, Figures 3–5 contain the patterns of SL1 group and the patterns of the adult in task 1, task 6, and in the post intervention task, respectively; Figures 6–8 contain the patterns of SL2 group and the adult’s ones in the same tasks. Within each figure, the patterns of the participants appear organized increasingly based on the suitability that the criterion behavior implies for the resolution of the task. Therefore, those patterns of the participants whose criterion behavior is less suitable are presented first and then those in which their criterion behavior is more appropriate. Adult patterns are presented starting with those whose criterion behavior implies more direct adult help to the participants and ending with those who assume less direct adult help. Figures 9–11 show the comparison of patterns obtained in each task for each group.

To facilitate the jointly reading of the patterns described and its representation in the Figures we indicate at the beginning of each paragraph the code and name of the criterion behavior that generated the patterns described.

Figures 3–5 show that in SL1 group the categories of participants considered as criterion behavior that have generated patterns occurring alone and/or co-occurring with other categories are: *Wrong use of strategy* (*UesEr*), *Change to a wrong strategy* (*CesEr*), *Correct use of strategy* (*UesC*), *Change to a correct strategy* (*CesC*), *Regulation* (*Rg*), *No error detection* (*Nde*), *Error self-detection* (*Ade*), and *Adjusted evaluation* (*Eva*). The categories of participants considered as criterion behavior that have not generated patterns are: *No response* (*NR*), *Unrelated behavior* (*Ds*), *Cheking break* (*Ic*), and *Non adjusted evaluation* (*Ena*).

*UesEr* (*Wrong use of strategy*): We begin by presenting the patterns generated by the criterion behavior less suitable for the resolution of the tasks: *Wrong use of strategy* (*UesEr*). It is appreciated that these patterns appear in the three tasks. In task 1 (Figure 3), *UesEr* generates three prospective patterns and one retrospective pattern. This retrospective pattern indicates that children use wrongly a strategy (*UesEr*) despite the fact that the adult previously offers them a concrete guide that points and directs them toward the adequate resolution of the activity (*Direct help* -*Adp*- in lag −1). Previously, participants execute more *Wrong use of strategy* (*UesEr*): in lag −2, *UesEr* co-occurs with verification behaviors (*Regulation* -*Rg*-) and incorrect evaluation behaviors (*Non adjusted evaluation* -*Ena*-) which hamper the participants from being able to change the type of response given; in lag −3, *UesEr* occurs in isolation. Participants persist executing these wrong actions despite the adult offers them more and different helps: help to understand the activity (*Repetition* -*Rep*- in lag −4) and, again, help to execute it (*Direct help* -*Adp*- in lag −5). In brief, this retrospective pattern indicates that *Wrong use of strategy* (*UesEr*) is a repetitive behavior in the action of participants despite the numerous adult helps. Prospective patterns indicate that a more direct and explicit adult intervention such as *Error correction* (*Adc*) is necessary for participants to perform correct strategies (either *Change to a correct strategy* -*CesC*- or *Correct use of strategy* -*UesC*-). In task 6 (Figure 4), this criterion behavior *UesEr* only generates three prospective patterns. After *UesEr*, the adult offers an *Indirect help* (*Ayi*). This help can sometimes be effective and can be followed by correct answers from the participants (*Change to a correct strategy* -*CesC*-). However, other times this adult help cannot be effective and then: (a) children can continue executing incorrect answers (more exactly, they can commit errors and do not detect them: *Change to a wrong strategy* with *No error detection* -*CesErNde*-) or (b) adult can offer more direct help to participants (*Error correction* -*Adc*-). In the post intervention task (Figure 5), this criterion behavior *UesEr* generates a single prospective and retrospective linear pattern, which implies a lower variability of child behavior. The pattern is prospective and retrospectively similar, as the participants can perform both *Change to a correct strategy* (*CesC*) and *Change to a wrong strategy* (*CesEr*). It is important to note that when errors occur, participants are always able to self-detect them for themselves (*Ade, CesErAde*). In addition, it stands out that prospectively, participants can perform all these skills autonomously, without adult help.

Therefore, as the intervention progresses and after its completion: (a) *Wrong use of strategy* (*UesEr*) is accompanied by a less adult intervention (some of these adult helps being gradually less explicit), until it can sometimes disappears in the post intervention task; (b) in addition, in this task, the participants themselves are able to self-detect their mistakes (*Ade*); aspect absent in tasks 1 and 6, and therefore implies progress in their planning skills; (c) the patterns are stabilizing, that is, there is not so much diversification of behaviors, as at the beginning of the intervention several patterns appear and in the post intervention task only one. This implies that *Wrong use of strategy* (*UesEr*) is decreasing, which also implies improvement, although it does not disappear. Therefore, there is still a possibility of progress in child behavior in this regard, although in turn we cannot forget the significant gain produced in terms of the self-detection of mistakes (*Ade*).

*UesErRg* (*Wrong use of strategy* co-occurring with *Regulation*): This criterion behavior generates patterns in tasks 1 and 6, but not in the post intervention task. In task 1 (Figure 3), *UesErRg* generates three prospective patterns and four retrospective patterns. However, prospective patterns are very brief (binary patterns) and are mainly made up of adult behaviors (*Error correction* -*Adc*-; *Direct help* -*Adp*-; *Indirect help* -*Ayi-*), without containing appropriate behavior of the participants (*No error detection* -*Nde*- is the only category appearing for participants). Therefore, these aids do not favor the emergence of appropriate child behaviors with greater force than those expected by chance. In retrospective patterns, adult intervention is also frequent, both to assist participants in understanding the task (*Command* -*Ord*- and *Repetition* -*Rep*-) and in its execution (*Indirect help* -*Ayi*-). Despite these aids, participants are not able to make correct answers, persisting in *Wrong use of strategy* (*UesEr*), sometimes accompanied by regulatory behaviors (*Rg*) but other times without detecting their error (*Nde*). In task 6 (Figure 4), *UesErRg* generates a single prospective and retrospective linear pattern. Prospectively, it is necessary adult *Direct help* (*Adp*) to participants change their strategy and give a correct answer (*CesC*) accompanied by regulatory behaviors (*Rg*). Retrospectively, participants use autonomously and correctly a strategy (*UesC*), also accompanied by regulatory behaviors (*Rg*). Therefore, there is a gain comparative to task 1, because the participants are able to execute correct behaviors (*UesC* and *CesC*), although sometimes they need adult help (*Adp*) to get it.

*CesErNde* (*Change to a wrong strategy* with *No error detection*): This criterion behavior only produces a pattern in task 6 (Figure 4). It is a single prospective pattern in which participants, thanks to *Direct help* from the adult (*Adp*), can change again their strategy and give a correct answer (*CesC*).

*CesErAde* (*Change to a wrong strategy* with *Error self-detection*) generates a pattern in the post intervention task (Figure 5), both prospectively and retrospectively. It is a linear pattern. The appearance of *Error self-detection* (*Ade*) (comparative to *No error detection* -*Nde*- that appears in task 6, and which has been described in the previous paragraph) implies progress. That is, although in both tasks the participants make mistakes, in task 6 they do not detect them and in the post intervention task they do. However, this *Error self-detection* does not entail an immediate correction of the error by the participants, as it is followed by adult help (*Repetition* -*Rep*- in lag +2) and *Wrong use of strategy* (*UesEr* in lag +3) until the participants execute correct actions (*CesC* in lag +5).

*UesC* (*Correct use of strategy*) generates patterns in all three tasks, being in all cases prospective patterns, never retrospective patterns. In task 1 (Figure 3) a binary pattern appears combined with an intervention of the adult (*Motivating help* -*Aym*-), although this adult behavior does not favor other child behavior. In task 6 (Figure 4) a binary pattern appears in which *Correct use of strategy* (*UesC*) leads to another *Correct use of strategy* (*UesC*). In the post intervention task (Figure 5) a pattern appears in which *Correct use of strategy* (*UesC*) implies that the adult performs *Motivating help* (*Aym*), then the participants continue with *Correct use of strategy* (*UesC*). Therefore, there is progress between task 1 and task 6 because in task 1, despite the help of the adult, no response was obtained from the participants, while in task 6, *UesC* is followed by another *UesC* autonomously, without requiring adult participation. However, after a period of non-intervention (post intervention task) it is appreciated that these skills are not consolidated as participants need adult intervention to maintain *Correct use of strategy* (*UesC*).

*UesCRg* (*Correct use of strategy* co-occurring with *Regulation*): Its capacity to generate patterns increases as the intervention progresses, as it does not generate patterns in task 1 but does in task 6 (Figure 4). This capacity to generate patterns is maintained 1 month after the end of the intervention as *UesCRg* behavior also generates a pattern in the post intervention task (Figure 5). The patterns that are generated in task 6 (Figure 4) are 1 prospective and 2 retrospective ones. In the latter (retrospective patterns) *Indirect help* of the adult (*Ayi*) prevails while in the prospective one *Adjusted evaluation* (*Eva*) appears in several lag. Despite the important progress of this *Eva* behavior, its use does not seem to be consolidated since it does not appear in the post intervention task (Figure 5). In this task, retrospectively, *Indirect help* of the adult (*Ayi*) is still necessary to maintain *Correct use of strategy* (*UesC*) with *Regulation* (*Rg*). Prospectively, adult help (in this case *Motivating help* -*Aym-*) is also necessary for the execution of these behaviors. Therefore, in the post intervention task, participants need adult help, of one kind or another, to use their strategies correctly and with regulation.

*CesC* (*Change to a correct strategy*): It does not generate patterns in task 1 (Figure 3), but it does in the other two tasks. In both tasks, it generates a prospective and retrospective linear pattern that highlights the following comments. Retrospectively, in task 6 (Figure 4), participants are able to *Change to a correct strategy* (*CesC*) due to having received a direct adult help (*Adp*) to replace their previously erroneous strategies (*UesEr*). However, in the post intervention task (Figure 5), the adult help disappears. The children can autonomously *Change to a correct strategy* (*CesC*) since they are able to self-detect the errors (*Ade*) they have previously performed (*UesEr*); this implies progress in their planning skills.

*Ade* (*Error self-detection*): This criterion behavior only generates patterns in the post intervention task (Figure 5). It does it both alone and co-occurring with *CesEr* (this pattern had already been commented previously). In addition, it is the only task in which this category *Ade* also appears as part of the patterns generated by other criteria behaviors. Therefore, the appearance of *Error self-detection* (*Ade*) in the post intervention task represents a progress in the planning processes of SL1 group participants. Also, in most of the patterns in which *Ade* appears (either generating patterns or forming part of others) adult help no appears. Therefore, *Ade* favors a more autonomous activity in children.

*Eva* (*Adjusted evaluation*): It only generates one pattern in task 6 (Figure 4). It is a retrospective pattern. For children to perform a result and evaluate it correctly (*Eva*) they have previously used correct strategies in a regulated way (*UesCRg* in lag −1, lag −3 and lag −5). These correct behaviors have been favored by adult *Indirect help* (*Ayi* in lag −2 and lag −4). It is noteworthy that *Adjusted evaluation* (*Eva*) appears in task 6 not only generating patterns but also forming part of other patterns. In contrast, in task 1, it does not appear in any pattern. Therefore, the *Adjusted evaluation* (*Eva*) does not appear in the patterns corresponding to the start of the intervention but does in those referring to its completion (also generating a pattern by itself), representing progress in the skills of the participants. However, this progress is not consolidated as in the post intervention task (Figure 5) *Adjusted evaluation* (*Eva*) it does not generate a pattern nor is it part of other patterns.

In summary, the patterns of SL1 group indicate that these participants have advanced in their planning skills throughout the intervention and after it, given that: (a) less and less adult help is required to participants perform correct behaviors; (b) the patterns generated by *Wrong use of strategy* (*UesEr*) (both occurring alone and co-occurring with other behavior) are decreasing; and although they do not disappear, the behaviors referring to *No error detection* (*Nde*) that appeared in some patterns during the intervention are replaced by *Error self-detection* (*Ade*) 1 month after its completion; (c) in task 6 *Change to a wrong strategy* (*CesEr*) generates a pattern by co-occurring with *No error detection* (*Nde*) while in the post intervention task it is co-occurring with *Error self-detection* (*Ade*); (d) *Adjusted evaluation* (*Eva*) appears for the first time in task 6, both generating a pattern and forming part of other patterns; (e) in the post intervention task the participants are able to detect by themselves their errors (*Ade*) and replace them with correct strategies (*Change to a correct strategy* -*CesC*-), even without needing the help of the adult.

Regarding the patterns generated by adult behaviors in SL1 group (Figures 3–5), the following is observed. Only the criteria behaviors *Direct help* (*Adp*) and *Indirect Help* (*Ayi*) generate patterns.

*Adp* (*Direct help*) generates patterns in task 1 (Figure 3) and in task 6 (Figure 4), but not in the post intervention task (Figure 5). Therefore, in this last task, the category’s capacity to generate patterns has decreased, which implies that 1 month after the intervention the participants are able to be more autonomous in their actions. However, their autonomy is not total due to other adult behaviors (*Indirect Help* -*Ayi-* and *Motivating help -Aym-*) appear even in this last task (post intervention task).

*Ayi* (*Indirect help*): This adult category generates patterns in the three tasks. In task 1 (Figure 3) and in task 6 (Figure 4), these patterns contain children’s actions both adequate (*Change to a correct strategy -CesC-; Correct use of strategy* with *Regulation -UesCRg-; Adjusted evaluation -Eva-*) and/or inadequate (*Wrong use of strategy* with *Regulation -UesErRg-; Wrong use of strategy -UesEr-; Change to a wrong strategy* with *No error detection -CesErNde-*). Nevertheless, in the post intervention task (Figure 5), these patterns contain only adequate children’s actions (*Correct use of strategy -UesC-; Correct use of strategy* with *Regulation -UesCRg-*). Hence, effectiveness of *Indirect help* (*Ayi*) is greater after the end of the intervention.

Figures 6–8 contain the patterns of SL2 group in tasks 1, 6 and post intervention task, respectively; as well as the patterns generated by adult behaviors with this group and in those tasks.

In SL2 group, the categories of participants considered as criterion behavior that have generated patterns occurring alone are: *Wrong use of strategy* (*UesEr*), *Change to a wrong strategy* (*CesEr*), *Correct use of strategy* (*UesC*), and *Change to a correct strategy* (*CesC*). In addition, the categories *Correct use of strategy* (*UesC*) and *Change to a correct strategy* (*CesC*) have generated patterns co-occurring with the category *Regulation* (*Rg*). The categories of participants considered as criterion behavior that have not generated patterns are: *No response* (*NR*), *Unrelated behavior* (*Ds*), *Checking break* (*Ic*), *No error detection* (*Nde*), *Error self-detection* (*Ade*), *Non adjusted evaluation* (*Ena*), and *Adjusted evaluation* (*Eva*).

*UesEr* (*Wrong use of strategy*): This criterion behavior is the least suitable for the resolution of tasks. It generates a pattern in tasks 1 and 6 but not in the post intervention task. In task 1 (Figure 6), it generates a linear prospective pattern in which, after *Direct help* of the adult (*Adp*), participants perform *Change to a correct strategy* (*CesC*). In task 6 (Figure 7), comparative to the execution in task 1, a progress is detected because: (1) correct behaviors (*CesC, UesC*) appear without adult intervention (adult help is not necessary for participants to replace their wrong strategies with correct ones); (2) furthermore, some of these participants’ correct behaviors are regulated (*Rg*), which implies that participants are able to verify their actions. In the post intervention task (Figure 8), *Wrong use of strategy* (*UesEr*) does not generate patterns, indicating a significant gain in the skills of the participants.

*CesEr* (*Change to a wrong strategy*): This criterion behavior only generates a pattern in task 6 (Figure 7). It is a prospective pattern. Despite adult help (*Repetition* - *Rep*-) the participants do not detect their errors (*Nde*) and persist in them (*Wrong use of strategy* -*UesEr*-).

*UesC* (*Correct use of strategy*) generates prospective and retrospective patterns in the three tasks (Figures 6–8). Prospectively, *Motivating help* (*Aym*) is always necessary, and/or *Indirect help* (*Ayi*) in the case of the post intervention task, so that the participants continue performing *Correct use of strategy* (*UesC*). Retrospectively, adult intervention is also necessary for participants to perform *Correct use of strategy* (*UesC*), although these aids are more direct at the beginning and at the end of the intervention than a month later: in task 1 (Figure 6), *Command* (*Ord*) appears and in task 6 (Figure 7), *Direct help* (*Adp*) appears, while in the post intervention task (Figure 8) *Motivating help* (*Aym*) and *Indirect help* (*Ayi*) appear. Therefore, 1 month after the intervention, the participants show more autonomy than at the beginning and at the end of the intervention, although they still require adult help.

*UesCRg* (*Correct use of strategy* co-occurring with *Regulation*) generates prospective and retrospective patterns in all three tasks. In tasks 1 and 6 (Figures 6 and 7, respectively) the patterns are very similar as, both prospectively and retrospectively, adult interventions of various types appear (*Motivating help* -*Aym*-; *Direct help* -*Adp*-; *Indirect help* -*Ayi*-; *Repetition* -*Rep*-; *Command* -*Ord*-) that allow participants to take correct actions (*UesCRg*, *CesCReg*, *CesC*). However, in the post intervention task (Figure 8) adult intervention is no longer necessary for participants to perform correct actions (*UesCRg*), which implies greater autonomy.

*CesC* (*Change to a correct strategy*) only generates patterns in task 6 and this happens both alone and by co-occurring with *Regulation* (*Rg*). The pattern generated by occurring alone is a prospective pattern showing that although at the end of the intervention the participants are able to correctly change and use a different strategy from the one previously used (*CesC*), they need adult help at various times (*Motivating help* -*Aym*- in lag +1 and *Indirect help* -*Ayi*- in lag +3) to continue to perform more correct actions (*UesCRg*). It stands out that these correct actions are accompanied by behaviors of *Regulation* (*Rg*), that is, verification behaviors to check that their action is correct.

*CesCRg* (*Change to a correct strategy* co-occurring with *Regulation*): It generates a prospective pattern very similar to that originated by itself alone (*CesC*), which has just been explained. This pattern, as already indicated, also appears in task 6 (Figure 7). This pattern generated by *CesCRg* is shorter than that originated by *CesC*, as fewer adult aids are needed (*Motivating help* -*Aym*- in lag +1) for the participants to continue to carry out more correct actions (these also with regulation -*UesCRg-*). Therefore, the comparison of the two patterns shows that the regulation performed by the children while they are changing their strategy (*CesCRg*) entails a lower need for adult help.

In summary, the patterns of SL2 group indicate that these participants have improved their planning skills throughout the intervention and after its completion because: (a) the patterns generated by both *Wrong use of strategy* (*UesEr*) and by *Change to a wrong strategy* (*CesEr*) disappear after the intervention; (b) the adult help children need to perform *Correct use of strategy* (*UesC*) is less directive (although it does not disappear), which implies greater autonomy for the children; (c) sometimes, adult help even disappears in the post intervention task (it occurs in the patterns generated by *Correct use of strategy* co-occurring with *Regulation* -*UesCRg-*) and children can perform more correct actions alone. This gain corroborates their greater autonomy.

Concerning the patterns generated by adult behaviors in SL2 group (Figures 6–8), the following is observed. The criteria behaviors *Direct help* (*Adp*) and *Indirect help* (*Ayi*) generate patterns. However, the criteria behaviors *Error correction* (*Adc*) and *Motivating help* (*Aym*) do not generate patterns.

*Adp* (*Direct help*) generates a pattern in task 1 and task 6 (Figures 6 and 7), favoring participants to make correct answers (*CesC*, *UesCRg*, *CesCRg*, *UesC*). However, *Adp* does not generate a pattern in the post intervention task (Figure 8). Therefore, in this task the participants show more autonomy and do not require adult directive assistance; which indicates progress in their planning skills.

*Ayi* (*Indirect help*) generates patterns in all three tasks (Figures 6–8). Its effect on the action of the participants is similar to the other adult help (*Direct help* -*Adp*-) because it also allows them to perform correct actions (*UesC*, *UesCRg*, *CesC*). However, *Ayi* continues generating patterns in the post intervention task (Figure 8). Therefore, this result (generation of patterns by *Indirect help* -*Ayi*- in the three tasks) together with that previously explained (generation of patterns by *Direct help* -*Adp*- in task 1 and 6 but not in the post intervention task) allows for the following conclusion: although the participants show more autonomy in the post intervention task than in task 1 and 6, they are not yet totally autonomous in their behavior.

The category *Motivating help* (*Aym*), despite not generating patterns in any of the three tasks, does appear in all of them as part of other patterns (Figures 6–8). This corroborates our previous statement: the participants have gained autonomy but are not yet totally autonomous in their behavior.

Comparing the patterns obtained by SL1 and SL2 groups throughout the intervention and after its completion (Figures 9–11), the following is observed: (1) both groups have progressed; (2) however, this progress is qualitatively different as it affects distinct planning skills: (2a) SL1 group progresses in the use of evaluation skills (*Ena* and *Eva*) and *Error self-detection* (*Ade*) (marked in bold and underlined in Figures 9–11). In task 1 (Figure 9), this group performs evaluations, but incorrect ones (*Non adjusted evaluation* -*Ena*-). At the end of the intervention (task 6; Figure 10) these evaluations are correct (*Adjusted evaluation* -*Eva*-), and they appear generating a pattern, but also forming part of other patterns. These evaluations are not maintained 1 month after the end of the intervention (post intervention task; Figure 11). However, in this post, intervention task for the first time *Error self-detection* (*Ade*) appears, both generating patterns and being part of others. So, *No error detection* (*Nde*) that appears in task 1 and task 6 is replaced by *Error self-detection* (*Ade*) in the post intervention task (marked in bold and underlined in Figures 9–11). All this allows us to conclude that SL1 group progresses in complex planning skills. (2b) In contrast, SL2 group presents changes only in more basic planning skills: *Wrong use of strategy* (*UesEr*), *Change to a wrong strategy* (*CesEr*), *Correct use of strategy* (*UesC*) and *Change to a correct strategy* (*CesC*). Specifically, incorrect behaviors (both *Wrong use of strategy* -*UesEr*- and *Change to a wrong strategy* -*CesEr-*) disappear 1 month after the end of the intervention (post intervention task; Figure 11). Correct behaviors (*UesC* and *CesC*) are performed with less adult help. At the beginning of the intervention (Figure 9) the adult’s continuous help is necessary for participants to carry out these correct behaviors (*UesC* and *CesC*) (aspect marked in bold and underlined in Figure 9). At the end of the intervention (Figure 10), in some cases, they can occur without adult help (aspect marked in bold and underlined in Figure 10). One month after the end of the intervention (Figure 11), *Correct use of strategy* co-occurring with *Regulation* (*UesCRg*) generates a long pattern without adult intervention (pattern marked in bold and underlined in Figure 11). Complex planning skills (*Non adjusted evaluation* -*Ena*-; *Adjusted evaluation* -*Eva*-; *Error* s*elf-detection* -*Ade*-) do not appear in this group in any of the three tasks.

## Discussion

There were two objectives of this study: (1) to show that the mixed methods approach can be useful in studying planning skills of children with ASD during and after an educational intervention; (2) to assess whether the planning skills of two groups of children with ASD (grouped according to their SL) improved during the intervention and if this progress was maintained 1 month after the end of the intervention.

Regarding objective 1, the mixed methods approach used in this work has allowed us to study in a rigorous and objective way the planning skills of children with ASD during and after the intervention. The first QUAL phase that constitutes the mixed methods approach have allowed us to address a pending challenge in the investigation of planning skills in ASD: create and implement an assessment instrument within an intervention capable of obtaining, in a natural and objective way, observational data on the functioning of the person. All that without altering the interactions that arise between children and adults in the dynamics of the intervention or adding extra evaluation elements that overload both (Gioia et al., 2010). In this sense, the constructed observation instrument (Figure 1) is a tool that can be of great help for future researchers and professionals in the evaluation of planning skills in children with ASD. Subsequently, the QUAN phase of the mixed methods approach followed. It implied to obtain the measurement parameters, to test the quality of the coded observational data and to carry out its analysis. Our initial observational dataset was qualitative but was transformed into quantitative data using lag sequential analysis (a quantitative analysis technique suitable for qualitative data). Since our initial dataset contained information not only about the primary parameter of frequency but also about other essential primary parameter, such as order (Bakeman, 1978; Anguera et al., 2001; Bakeman and Quera, 2011; Quera, 2018), quantification was robust. The consideration of the order parameter provided us with the means to know the different components of sequences of behavior as it occurred. Thereby, the order parameter was crucial for detecting hidden structures through the quantitative analysis of relations among different codes in our initial observational dataset. However, before carrying out this analysis, it was necessary to check the quality of the observational data. In this study, the quality of observational data was verified through intra- and inter-observer reliability. They were computed through Cohen’s kappa coefficient. All results showed a very good agreement. So, we could conclude that the observational data obtained was excellent. Consequently, we could analyze them. As we have already indicated, data analysis was conducted using a particularly fitting technique for analyzing complex human behaviors in order to obtain detailed sequences of behaviors: lag sequential analysis. This technique offered patterns of behavior that inform the sequential structure of planning skills performed by children in interaction with the educational specialist. Thereby, the mixed methods approach has allowed to capture a large amount of invaluable information through other methodologies. In most of the research carried out with participants with ASD that try to analyze the effects of interventions on different areas of their development other methodologies (especially, selective methodology) are used. However, data obtained in these methodologies do not inform the changes and differences produced between the beginning and the end of the intervention (Kasari et al., 2014). In contrast, mixed methods approach used in this study allows to capture these changes and differences. Finally, we returned to QUAL phase of the mixed methods approach with the interpretation of the patterns behavior, permitting seamless integration. To do so, we considered the objectives of our study and prior researches. So, we could conclude that the two groups of children with ASD improved their planning skills during and after the intervention. (We explain this aspect more deeply later since it is closely linked to our objective 2).

In short, the mixed methods approach adopted has shown its enormous potential to help us to study the improvement of the planning skills of children with ASD, and consequently, to improve their quality of life; aspect that proves to be particularly deficient in children and adolescents with ASD compared to their typically developing peers (de Vries and Geurts, 2015). Therefore, and in accordance with other authors (Arias-Pujol et al., 2015; Rodríguez-Medina et al., 2016, 2018; Alcover et al., 2019), we advocate the use of mixed methods approach. The use of the mixed methods approach (and, more exactly, the observational methodology considered in itself as mixed methods), due it offers rigor and flexibility, is still more necessary and useful when it comes to the assessment of participants of a young age and special characteristics, as is the case of this study (Anguera, 2003).

Regarding objective 2, the results obtained indicate that the intervention involved gains for both groups of participants since the two groups improved their initial planning skills. Some of these gains were common for both groups and others specific to SL1 group. Both groups: (1) made fewer mistakes (*UesEr* and *CesEr*); (2) performed more correct executions (*UesC* and *CesC*). Both aspects (1 and 2) imply that the participants’ behavior was adjusting to the demands of the task; (3) acquired greater autonomy in their actions: the application of scaffolds during the intervention are gradually less directive and once the intervention finished, participants became more proficient and autonomous. In addition to these gains, SL1 group was also able to: (a) self-detect their mistakes (*Ade*) and (b) evaluate properly their action (*Eva*); sometimes even being able to do it autonomously. Since both types of skills (*Ade* and *Eva*) constitute complex planning skills (Zelazo et al., 1997; Ward and Morris, 2005), it can be said that, after the intervention, SL1 group came to use complex planning skills autonomously. However, SL2 group never used complex planning skills (with or without the help of the adult), which entails negative consequences in the resolution of tasks, since effective planning implies a cyclical and continuous process in which the self-detection of errors and the evaluation of performance and results are required, in addition to other skills (Hill, 2004a; Olde Dubbelink and Geurts, 2017).

In summary, the results obtained allow us to conclude that this research has led to a breakthrough in terms of the numerous possibilities of practical application offered by the mixed methods approach as well as an advance in the very scarcely studied field of planning skills in ASD (Olde Dubbelink and Geurts, 2017).

The intervention has produced positive effects in both groups of participants. The literature review indicates that executive functions and in particular planning skills in children with ASD show an atypical development trajectory. Without intervention, their executive deficits persist throughout their life cycle, remaining below the performance of their standard developing peers (Pellicano, 2010). Hence the importance of carrying out research such as described here, to understand the improvement produced by different interventions on executive functions in children with ASD.

This is also precisely one of the positive aspects of this work: focus on one of the executive function deficits in children with ASD such as planning. The majority of studies, and even more of the interventions, are focused on the core deficits of ASD (especially, difficulties in social communication and interaction), forgetting other problems that affect these people and their quality of life (Bond et al., 2016).

Despite the progress shown by the two groups of participants after the intervention, the results obtained in this study indicate that both are susceptible to continue improving their planning skills. Therefore, it is recommended to incorporate some improvements into the intervention: (1) to prolong the intervention over time by increasing the number of tasks and activities in order to SL1 group continue progressing and strengthening their complex planning skills and SL2 group begin to use them; (2) to provide a more specific scaffolding adjusted to the level of development shown by the participants, especially those of SL2 group. This would imply modifying the support of the adult to guide the tasks, incorporating the systematic teaching of a series of strategies that cognitively enhance more complex behaviors in both groups of children (Meinchenbaum, 1974); (3) to increase the number of activities in the post intervention task; (4) to establish another subsequent post intervention measurement point to see if the effects of the intervention are maintained for a longer period. It could be 3 or 6 months later the post intervention task; (5) to design activities and tasks to try to extend the benefits of the intervention to other tasks similar to those training tasks used during the intervention (near transfer), as well as transfer to behavior on less similar non-trained tasks (far transfer) (Zelazo et al., 2017).

It should not be forgotten that the sample of this study is small. Therefore, the improvement observed in the two groups of participants should be taken with caution. In this sense, it would be necessary to have a larger sample in order to obtain a greater amount of data that support these findings. The small sample size was due to the inherent complexity involved in any intervention with this type of population, together with the difficulties of accessing a larger number of participants. In relation to this last aspect, it would be a challenge to promote models of collaboration between educational and research centers that foster practical research focused on innovative pedagogical strategies, as well as evidence-based interventions to improve the quality of educational practice in the field of autism (Locke et al., 2016; Stahmer et al., 2017).

Although, as we have already mentioned, this study shows the improvement of planning skills of each group both during and after the intervention, we consider it would be interesting to carry out an individual analysis of the records of each participant in the future. This would allow us to obtain a greater knowledge of the progress that each one of them was achieving, and therefore, adjust the intervention more to their needs.

Given the relevance of executive functions for integral development and adequate adaptation to daily life, and consequently, the great obstacle that their executive deficits involve to people with ASD, we believe that in the future other interventions should be designed to improve different executive components such as flexibility, inhibition, etc. This study and its suggestions for improvement indicated previously, could contribute in this area.

On the other hand, at the methodological level and more exactly in relation to data analysis, in the future, this study could be complemented by including other observational data analysis techniques different from the one used here (lag sequential analysis), such as *polar coordinate analysis* (Sackett, 1980; Anguera et al., 2018b) and *temporal pattern* (*T-patterns*) *detection* (Magnusson et al., 2016). We are unaware of works in which these data analysis techniques have been used for the evaluation of planning skills in children with ASD. Although the specifics of each of these three observational data analysis techniques (lag sequential analysis, polar coordinate analysis, and T-pattern detection) differ, all of them allow to analyze and increase understanding of the internal structure of observed behavior, as evidenced by the only two works we know that have applied these three techniques together for the analysis of child behavior, and also in school context (Santoyo et al., 2017; Escolano-Pérez et al., 2019). Consequently, the complementary use of these three powerful data analysis techniques would allow a more exhaustive evaluation of the planning skills of children with ASD.

## Data Availability Statement

The raw data supporting the conclusions of this article will be made available by the authors, without undue reservation, to any qualified researcher.

## Ethics Statement

Research was evaluated and approved by the Education Doctoral Program Academic Commission of Zaragoza University. Research was also approved by the school management teams. In accordance with the Organic Law 15/1999 of December of Protection of Personal Data (1999, BOE n_ 298 of December 14) all the parents of the participants signed the informed consent authorizing the participation of their children in the study and being recorded while playing. In addition, and following the guidelines of the aforementioned law, the observers signed the confidentiality agreement. No ethics special approval was required for this research since the Spanish public education system and national regulations require no such approval. Each participant received a small reward (two chocolates) in gratitude for their participation.

## Author Contributions

EE-P involved in conceptual and methodological structure, literature review, systematic observation, drafting of the manuscript, results and discussion. MA-F involved in collecting data, systematic observation, literature review and drafting of the manuscript and also contributed to results and its discussion. MH-N involved in methodological structure, data analysis, results and discussion. All of the authors contributed to revising the manuscript and provided final approval of the version to be published.

### Conflict of Interest

The authors declare that the research was conducted in the absence of any commercial or financial relationships that could be construed as a potential conflict of interest.

The handling editor declared a past collaboration with one of the authors EE-P.

## Footnotes

^1^http://www.ub.es/comporta/sg/sg_s_download.htm
